# Higher-order dynamic effects of uncertainty risk under thick-tailed stochastic volatility

**DOI:** 10.1186/s40854-022-00370-5

**Published:** 2022-06-07

**Authors:** Xiao-Li Gong, Jin-Yan Lu, Xiong Xiong, Wei Zhang

**Affiliations:** 1 School of Economics, Qingdao University, Qingdao, 266061 China; 2 College of Management and Economics, Tianjin University, Tianjin, 300072 China; 3 China Center for Social Computing and Analytics, Tianjin, 300072 China

**Keywords:** Uncertainty risk, High-dimensional DSGE, Epstein–Zin preferences, Stochastic volatility, Thick tail distribution

## Abstract

Sudden and uncertain events often cause cross-contagion of risk among various sectors of the macroeconomy. This paper introduces the stochastic volatility shock that follows a thick-tailed Student’s *t*-distribution into a high-order approximate dynamic stochastic general equilibrium (DSGE) model with Epstein–Zin preference to better analyze the dynamic effect of uncertainty risk on macroeconomics. Then, the high-dimensional DSGE model (DSGE-SV-*t*) is developed to examine the impact of uncertainty risk on the transmission mechanism among macroeconomic sectors. The empirical research found that uncertainty risk generates heterogeneous impacts on macroeconomic dynamics under different inflation levels and economic states. Among them, a technological shock has the strongest impact on employment and consumption channels. The crowding-out effect of a fiscal policy stimulus on consumption and private investments is relatively weakened when considering uncertainty risk but is more pronounced during periods of high inflation. Uncertainty risk can partly explain the decline in investments and the increase in interest rates and employment rates, given the impact of an agent’s risk preferences. Compared with external economic conditions, the inflation factor has a stronger impact on the macro transmission mechanism caused by uncertainty risk.

## Introduction

The sudden impact of COVID-19 in 2020 has brought uncertainty risks to global economic activities. Given the negative impact of the epidemic, the interweaving of multiple risk factors has brought uncertainties to economic development (Szczygielski et al. 2021). As Chinese president Xi Jinping emphasized, “In the coming period, we will face more unfavorable external environments and should be prepared to deal with a series of new risks and challenges.” The present downward pressure on various countries’ economies is increasing, and the risk of sudden uncertainty has caused a short term, rapid recession in the macroeconomy (Bloom 2009). In an economic downturn, the public’s expectations of increasing uncertainties and adverse shocks in the future have increased. The general consensus is that government departments need to take effective responsibility and adopt effective policies to rescue the market. Uncertainty can create business cycle fluctuations, and the public’s expectations of uncertainty will prompt regulatory authorities to frequently introduce macrocontrol policies to boost the economy, which will exacerbate the risk of uncertainty.

In the context of the “financialization” of real enterprises, uncertainty risk often leads to unemployment and a decline in output and investments. Determining how uncertainty risk superimposed on the intervention of government departments and the expected preferences of investors affects enterprises’ investment behavior is necessary to prevent and dissolve public risks—an important public finance function. Given the current background that all countries place importance on consumption, investment, employment, and expectations, the answer to this question is that high-quality economic development is needed.

What impact will uncertainty risk have on the transmission mechanism of the aforementioned macroeconomic variables? Previous studies mostly analyzed the impact of sudden shocks on the business cycle from the perspective of exogenous shocks but failed to accurately measure the risk of sudden uncertainty. Clarifying how uncertainty risk represented by the COVID-19 epidemic affects the activities of macroeconomic entities will assist with economic recovery and policy analysis in the postepidemic era. Of great significance for expectation management is measuring the macrovolatility effect caused by uncertainty risk and exploring the transmission mechanism between uncertainty and the macroeconomy.

## Literature review

Uncertainty risks caused by sudden event shocks represent incalculable economic losses from emergencies without any warning and are random in nature. Uncertainty risks can cause endogenous disasters in the macroeconomy (Petrosky-Nadeau et al. 2018), making the tail risks created by sudden uncertainties contagious among various macroeconomic sectors. Ludvigson et al. (2020) pointed out that the COVID-19 pandemic has taken a toll on capital markets and the household sector’s labor, and the macro uncertainty created will last for a long time. Guerrieri et al. (2020) pointed out that supply shocks caused by the COVID-19 pandemic can cause a demand-deficiency recession. In 2020, the global economy—affected by the COVID-19 pandemic—experienced a significant downturn, and the U.S. stock market has rarely experienced continuous circuit breakers (Baker et al. 2020). Uncertainty is an important source of macroeconomic volatility and can characterize the risks not captured by volatility (Chung and Chuwonganant 2014). The economic uncertainty resulting from COVID-19 has become one of the biggest threats to global financial markets (Aslam et al. 2020; Sharif et al. 2020). Nigmonov and Shams (2021) found pandemic-induced exposure to default risk in the marketplace lending market that can increase tail risk contagion channels in the international financial system (Guo et al. 2021).

Previous studies on the impact of extreme risks mostly focused on the financial market sector, especially the hedging strategies for market volatility in extreme situations (Racicot et al. 2021; Racicot and Théoret 2022). The dynamic shock responses for macroeconomic sectors affected by extreme risks are less studied. In addition, the use of nonstructural models (VAR, TVAR) to describe macroeconomic uncertainty often lacks microscopic foundations and cannot properly reflect the microscopic mechanisms and transmission channels of various macroeconomic sectors under extreme risk shocks. Most previous studies on macrouncertainty lacked a microfoundation; however, the dynamic stochastic general equilibrium (DSGE) model provides a microtheoretical foundation. This model can fully consider the impact of different shock factors, making it widely applied when investigating the volatility effects of unexpected shocks (Xiao 2018). Fernández-Villaverde et al. (2015a, b) used the DSGE model to analyze the impact of uncertainty on economic fluctuations, providing a research paradigm for such problems. Husted et al. (2020) employed the DSGE model to estimate the aggregate effects of shocks to monetary policy uncertainty on output, credit spreads, and other variables. Castelnuovo et al. (2018) utilized the New Keynesian DSGE model to study the real effects of monetary policy shocks characterized by high and low macroeconomic uncertainty on the real economy. However, most studies only focused on the impact of specific uncertainties and did not systematically account for the various characteristics of uncertainty risk, significantly reducing a model’s ability to portray disaster risks. Moreover, most previous studies that analyzed uncertainty often adopted a first-order perturbation method to approximate the DSGE model (Heiberger et al. 2017; Niu et al. 2018; Heiberger 2020). However, uncertainty risk exists in the third-order term of the DSGE model, which is related to the third-order disturbance approximation (Andreasen 2012). Therefore, a third-order approximation to the DSGE model is required to more accurately analyze the dynamic impact of uncertainty risk on the macroeconomic transmission mechanism.

Uncertainty risk is often viewed as being accompanied by sudden characteristics and stochastic volatility. Scholars including Justiniano and Primiceri (2008), and Diebold et al. (2017) gradually introduced stochastic volatility into the DSGE model (DSGE-SV). The suddenness of uncertainty risk can be characterized by the tail risk that it causes. The probability distribution of the exogenous shocks of macroeconomic variables is no longer the normal distribution when affected by uncertainty risk; instead, it exhibits thick-tailed characteristics (Gong et al. 2020). Therefore, both the thick-tailed, nonnormality feature of the sudden shock process and stochastic volatility characteristics must be considered when using the DSGE model to describe the dynamic macroeconomic effects of uncertainty risk (Alessandri and Mumtaz 2019). Unfortunately, given the complexity of the high-dimensional nonlinear DSGE model, previous studies did not incorporate both the thick-tailed nonnormal distribution characteristics and the stochastic volatility characteristics of uncertainty risk. This paper introduces the Student’s *t*-distribution and time-varying volatility into the exogenous shock of the third-order approximate DSGE model to better describe the dynamic macroeconomic effects caused by uncertainty risk statistically and develops the high-order approximate DSGE-SV-*t* model. Specifically, this paper introduces the thick-tailed distribution and stochastic volatility characteristics into the third-order approximate DSGE model for all exogenous shocks to reflect uncertainty risk. We then further analyze the impact of uncertainty risk on the transmission mechanism among macro variables. The constructed model can depict the stochastic volatility characteristics of macroeconomic tail risk contagion and the exogenous shocks caused by the suddenness of emergencies and is a better quantitative fit with the actual economic data. Unlike the previous DSGE stochastic volatility model,^1^ our high-dimensional model first makes a third-order approximation to DSGE to capture uncertainty risk.

Moreover, this paper adopts the pruned method of Andreasen (2018) when using the third-order method to approximate a high-dimensional DSGE model to avoid its exploding path. At the same time, given the development of the DSGE model enables it to present more parameters, endogenous variables, and exogenous shocks (Gertler 2015). Scholars mostly use Bayesian learning models for parameter estimation, such as those from Koop et al. (2013) and Fernández-Villaverde and Guerrón-Quintana (2021), to calibrate numerous parameters. The introduction of a nonnormal distribution and stochastic volatility characteristic has increased the model’s complexity. More advanced estimation methods are needed for such nonlinear high-dimensional DSGE models (Ruge-Murcia 2012). Although Fernández-Villaverde (2015a, b) used the Bootstrap particle filter method to estimate the DSGE model that contains stochastic volatility, the method suffers from particle degradation and reduces the accuracy of likelihood estimation when considering the thick tail factor. In addition, because uncertainty risk only exists under high-order approximation, the traditional Bayesian estimation method of Bianchi (2013) is no longer applicable. Fortunately, Chib and Ramamurthy (2010) proposed the random block Metropolis–Hastings (RB-MH) algorithm, which can be used to sample the posterior distribution of high-dimensional DSGE models. Using this method can significantly improve the estimation efficiency of high-dimensional DSGE models containing nonnormal and nonlinear latent variables (Kapetanios et al. 2019). Therefore, we construct a high-dimensional DSGE-SV-*t* model that includes the Student’s *t*-distribution and stochastic volatility feature in shocks. Moreover, the random block RB-MH algorithm is used to manage the estimation issues of the DSGE-SV-*t* model.

Most prior studies on uncertainty risk mainly focused on examining the effects of monetary policy. Few relevant studies exist on the macrocontrol effects of fiscal policy and the risk expectations of agents in this context. Luo (2019) pointed out that the invisible debt of local government agencies through financing platforms significantly affects the macro leverage ratio. Therefore, this paper also considers in the DSGE model the behavioral process of government departments, concentrating on an analysis of the regulatory role of fiscal policy given uncertainty risk. Meanwhile, the DSGE model with Epstein–Zin preference is used to reflect investors’ expectations of future uncertainty.

This paper aims to describe the dynamic effects of uncertainty risk represented by the COVID-19 epidemic on the macroeconomy. Previous studies found that uncertainty risk is often unmeasurable. However, we employ the difference between the third- and first-order effects of the DSGE model to accurately measure it. At the same time, uncertainty risks are divided into those with and without a suddenness nature. This paper innovatively introduces the Student’s *t*-distribution into the stochastic fluctuation shock of the DSGE model to measure uncertainty risks with a suddenness nature as represented by the COVID-19 pandemic and uses the thick-tailed distribution to statistically describe the suddenness characteristics of such risks.

The main contributions of this paper are as follows. (1) We introduce into a New Keynesian DSGE model with an Epstein–Zin preference the stochastic volatility shock that follows the thick-tailed distribution. (2) Then, the high-dimensional DSGE model (DSGE-SV-*t*) is developed to examine the impact of uncertainty risk on the transmission mechanism among macroeconomic sectors. (3) Considering that significant literature already exists on the effect of monetary policy given uncertainty risk, we primarily focus on analyzing the impact of uncertainty risk on macroeconomic transmission from the perspective of an agent’s risk preference and fiscal policy shocks. (4) In addition, we investigate the variations in macroeconomic transmission caused by the existence of uncertainty risk in different economic environments, such as different inflation levels and economic states. The research in this paper has important reference value for the supervision department in all countries when adopting rescue measures in disaster risk situations and to improve resilience.

## Methodology

### State space form of high-dimensional DSGE model

We extended the New Keynesian model to include thick-tailed structural shocks and time-varying volatility to better characterize the statistical properties of uncertainty risk. The thick-tailed structural shocks and time-varying volatility are achieved by specifying a Student’s *t*-error in stochastic volatility shocks. Because the constructed thick-tailed DSGE model with stochastic volatility contains high-order moments, it reflects agents’ expectations of the uncertainty of future shocks. Considering that the high-dimensional DSGE model has many endogenous variables, exogenous shocks, and nonlinear latent variables, expressing it in the form of state space is preferred, as follows.

1 $$\Gamma_{0} (\Theta^{S} )x_{t} = \Gamma_{1} (\Theta^{S} )x_{t - 1} + \Psi \varepsilon_{t} + \Pi \eta_{t}$$

where Θ^*S*^ represents the structural parameter vector, *x*_*t*_ represents the model variable vector, *ε*_*t*_ represents the structural shock vector, *η*_*t*_ represents the predictive error vector, and (Γ_0_, Γ_1_, Ψ, Π) represents the coefficient matrix.

Mumtaz and Theodoridis (2020) pointed out that, given the influence of uncertainty risk, the probability distribution of variables often follows a nonnormal distribution. Moreover, compared with the adverse shocks during the economic boom, agents tend to pay more attention to adverse shocks during an economic recession. Hence, following Chib and Ramamurthy (2014), we suppose that the innovation term *ε*_*t*_ of the shock follows a multivariate Student’s *t*-distribution to capture the thick-tailed distribution, namely, *ε*_*t*_ ~ *t*_*v*_(0, Σ_*t*_), where *v* is the degree of freedom and Σ_*t*_ denotes the diagonal matrix with time-varying volatility. A Gamma distribution is usually introduced to make the estimation convenient, and each element of *ε*_*t*_ is expressed as a normal mixture. The logarithm of volatility *h*_*t*_^*s*^ = *lnσ*_*s,t*_^2^ follows the stable dynamic process of stochastic volatility, where (*μ*_*s*_, *φ*_*s*_, σ_*s*_^2^) is the volatility parameter and is uniformly expressed as Θ^*V*^. Then, Eqs. (1)–(3) help express the constructed high-dimensional DSGE-SV-*t* model in the form of state space. Moreover, the specific settings of each sector of the DSGE model are as follows.

2 $$\varepsilon_{t}^{s} = \lambda_{t}^{ - 1/2} e^{{h_{t}^{s} /2}} \xi_{t}^{s} \;,\;\lambda_{t} \sim G(\nu /2,\nu /2),\;\;\xi_{t}^{s} \sim N(0,1)$$

3 $$h_{t}^{s} = (1 - \phi_{s} )\mu_{s} + \phi_{s} h_{t - 1}^{s} + \eta_{t}^{s} ,\;\;\eta_{t}^{s} \sim N(0,\omega_{s}^{2} )$$

### Model specification of high-dimensional DSGE model

We have extended the traditional DSGE model to incorporate heavy tail and stochastic volatility features to examine the impact of uncertainty risk on macroeconomic dynamics. The specification of the DSGE model is composed of four sectors, namely, households, manufacturers, government departments, and the central bank. Households optimize their consumption and labor supply given intertemporal budget constraints. Manufacturers are divided into final and intermediate product manufacturers and face perfectly competitive and monopolistic markets, respectively. Government departments and central banks carry out macroeconomic regulations to help the economy recover, through which government departments obtain fiscal revenues through taxation and bond issuances and the provision of subsidies to the unemployed.

### Households

Consider that all household sectors have an infinite lifetime, forming a continuum on the interval [0, 1]. Representative households maximize their lifetime utility by choosing consumption and labor supply. We assume that the consumption and labor levels of the household sector are, respectively, *C*_*t*_(*h*) and *N*_*t*_(*h*), and household preferences satisfy separability, as shown in Eq. (4). Rudebusch and Swanson (2012) pointed out that specifying the investor’s recursive Epstein–Zin preference in the DSGE model can improve the model’s empirical fit by separately specifying the risk aversion coefficient because the risk parameter of the Epstein–Zin preference can reflect the degree of agents’ aversion to increasing uncertainty. Therefore, we assume that economic agents have the following preference form in Eq. (5).

4 $$u(C_{t} (h),Z_{t} ,N_{t} (h)) = \frac{{(C_{t} (h) - bC_{t - 1} )^{{1 - \sigma_{C} }} }}{{1 - \sigma_{C} }} - \chi_{0} \left[ {ZZ_{t - 1} (\frac{{Z_{t - 1} }}{{Z_{t - 2} }})^{{\rho_{z} }} e^{{\sigma_{z} w_{z,t} }} } \right]^{{1 - \sigma_{C} }} \frac{{N_{t} (h)^{{1 + \sigma_{L} }} }}{{1 + \sigma_{L} }}$$

5 $$V_{t} (h) = u(C_{t} (h),Z_{t} ,N_{t} (h)) + \beta (E_{t} V_{t + 1} (h)^{1 - \gamma } )^{{\frac{1}{1 - \gamma }}}$$

where *σ*_*L*_ denotes the reciprocal of the Frisch elasticity, *σ*_*C*_ denotes the reciprocal of intertemporal substitution elasticity, *Z*_*t*_ denotes the productivity process, *b* is the habitual smoothing factor, *Z* is the steady-state value of productivity growth, and *ρ*_*z*_ and *σ*_*z*_ represent the degree of continuity and the standard deviation, respectively. *β* is the time discount factor, and *γ* denotes the risk aversion parameter, reflecting agents’ willingness to resolve the uncertainty of the expected consumption as soon as possible. *E*_*t*_ is the conditional expectation operator, and *E*_*t*_*V*_*t*+1_(*h*)^1–*γ*^ represents the risk aversion expectation operator that reflects agents’ degree of risk aversion.

The household sector provides differentiated labor services to intermediate product manufacturers. The comprehensive integration of different types of labor services *n*_*t*_(*h*) utilizes the Dixit–Stiglitz aggregator, and the overall labor services required by intermediate product manufacturers are identified in Eq. (6). Significant differences exist in the specification of the optimal nominal wage in existing research; in this paper, we adopt the pricing mechanism of Calvo (1983). Among the various differentiated labor services provided by the household sector, the proportion of wages that cannot be adjusted in each period is *m*_*ω*_. The nominal total wage is indexed on the basis of the previous nominal wage rate in accordance with the weighted average method of Eq. (7) (Leeper et al. 2017), as follows:

6 $$N_{t} = \left[ {\int_{0}^{1} {n_{t} (h)^{{\frac{1}{{1 + \eta^{\omega } }}}} dh} } \right]^{{1 + \eta^{\omega } }}$$

7 $$W_{t} = \left[ {(1 - m_{\omega } )(W_{t}^{o} )^{{ - \frac{1}{{\eta^{\omega } }}}} + m_{\omega } (\pi^{{1 - \chi^{\omega } }} \pi_{t - 1}^{{\chi^{\omega } }} )^{{ - \frac{1}{{\eta^{\omega } }}}} W_{t - 1}^{{ - \frac{1}{{\eta^{\omega } }}}} } \right]^{{ - \eta^{\omega } }}$$

where *η*^*ω*^ represents the shock of exogenous wages, *W*_*t*_ represents the nominal wage rate, *W*_*t*_^*o*^ is the optimal nominal wage rate, and *χ*^*ω*^ measures the degree of wage indexation.

The effective capital *K*_*t*_(*j*) is related to the physical capital stock *K*_*t–*1_^* s*^(*j*), *K*_*t*_(*j*) = *l*_*t*_(*j*)*K*_*t–*1_^* s*^(*j*), where *l*_*t*_(*j*) is the capital utilization rate selected by households. This utilization rate causes an adjustment cost *S*(*l*_*t*_) per unit of physical capital. Moreover, *l* = 1 when in the steady state, and we define the capital utilization parameter *κ* such that *S*″(1)/*S′*(1) = *κ*/(1 – *κ*). The accumulation process of physical capital in the household sector satisfies the following:

8 $$K_{t}^{s} (j) = (1 - \delta )K_{{t{ - }1}}^{s} (j) + [1 - {\rm B}_{i} (\frac{{I_{t} (j)}}{{I_{t - 1} (j)}})]I_{t} (j)$$

where *B*_*i*_(·)*I*_*t*_ denotes the investment adjustment cost. The investment adjustment coefficient *s* satisfies *B*_*i*_′(1) = *s*, and the investment cost also decreases when *s* decreases.

### Final goods producer

Suppose that two kinds of manufacturers exist in the production sector. The intermediate product manufacturers produce products *i* ∈ [0,1] and face a monopolistic competition market. The final product manufacturers use intermediate manufacturers’ products for processing and face a perfectly competitive market. The final product manufacturer buys the intermediate product *Y*_*t*_(*i*) from the intermediate manufacturer and engages in aggregate production in the form shown in Eq. (9). The final product manufacturer adopts profit maximization as the principle and obtains the demand conditions in Eq. (10) for the intermediate product by solving its profit maximization issue.

9 $$Y_{t} = \left( {\int_{0}^{1} {Y_{t} (i)^{{\frac{\eta - 1}{\eta }}} di} } \right)^{{\frac{\eta }{\eta - 1}}}$$

10 $$Y_{t} (i) = Y_{t} \left( {{{P_{t} (i)} \mathord{\left/ {\vphantom {{P_{t} (i)} {P_{t} }}} \right. \kern-\nulldelimiterspace} {P_{t} }}} \right)^{ - \eta }$$

where *η* represents the elasticity of substitution between differentiated intermediate products.

### Intermediate goods producer

Intermediate goods manufacturers employ labor and purchase or rent production materials for production. The production function of a single intermediate goods manufacturer is as follows:

11 $$Y_{t} (i) = A_{t} [K_{t} (i)]^{\alpha } [N_{t} (i)]^{1 - \alpha }$$

where *A*_*t*_ represents the total factor productivity, and *α* represents the capital production share.

Monopolistic competition exists among intermediate product manufacturers, given the differences in their production and management abilities. Therefore, intermediate product manufacturers need to set prices for their products. Following the traditional Calvo pricing method, this paper assumes that the intermediate product market experiences some price stickiness. That is, the proportion of firms that can reset prices in each period is assumed to be 1 − *m*_*p*_, the optimal price is *P*_t_^*^(*i*), and the proportion of firms that maintain the previous period’s price is *m*_*p*_. Then, the intermediate product manufacturer’s function of maximizing the economic benefit in the current period can be expressed as follows:

12 $$\begin{gathered} \max E_{t} \sum\limits_{j = 0}^{\infty } {M_{t,t + j} (\beta m)^{j} } \left[ {\frac{{P_{t}^{*} (i)\Pi_{t + j - 1}^{\iota } }}{{P_{t + j}^{*} (i)}} - MC_{t + j} (i)} \right]Y_{t + j} (i) \hfill \\ s.t.\;\;Y_{t + j} (i) = \left( {\mathop \prod \limits_{s = 0}^{j} \Pi_{t + s - 1}^{\iota } {{P_{t} (i)} \mathord{\left/ {\vphantom {{P_{t} (i)} {P_{t + j} }}} \right. \kern-\nulldelimiterspace} {P_{t + j} }}} \right)^{ - \eta } Y_{t + j} \hfill \\ \end{gathered}$$

where *ι* represents the price index, and *M*_*t*_ represents the random discount factor:$M_{t + 1} = \left( {\frac{{C_{t} (h) - bC_{t - 1} }}{{C_{t + 1} (h) - bC_{t} }}} \right)^{{\sigma_{C} }} \left( {\frac{{V_{t + 1}^{ - \gamma } }}{{(E_{t} V_{t + 1}^{1 - \gamma } )^{{{\gamma \mathord{\left/ {\vphantom {\gamma {\gamma - 1}}} \right. \kern-\nulldelimiterspace} {\gamma - 1}}}} }}} \right)$.

### Government sector and central bank

Government agencies provide funds for their interest payments and expenditures by issuing government bonds and taxing labor, capital, and consumption. In addition, the government exercises macrocontrol of the economy by implementing fiscal policies. The transfer payments between households are assumed to be identical, and the fiscal budget constraint satisfies Eq. (13). The fiscal rules include the response of fiscal instruments to debt/GDP ratios to ensure that they are sustainable. Additionally, the specific form of fiscal expenditure *G*_*t*_ and transfer payment *Z*_*t*_ is shown in Eq. (14).

13 $$P_{t} G_{t} + D_{t - 1} + (1 - N_{t} )P_{t} B_{t} = T_{t} + \frac{{D_{t} }}{{R_{t} }}$$

14 $$G_{t} = \rho_{g} G_{t - 1} - (1 - \rho_{g} )\alpha_{g} v_{t - 1} + \varepsilon_{g,t} ,\;\;Z_{t} = \rho_{z} Z_{t - 1} - (1 - \rho_{z} )\alpha_{z} v_{t - 1} + \varepsilon_{z,t}$$

where *G*_*t*_ = *g*_*t*_*Y*_*t*_ denotes government expenditure, *g*_*t*_ denotes the share of government expenditures in the national economy, *D*_*t*_ denotes government debt, *R*_*t*_ represents the returns from investing in government debt, *T*_*t*_ denotes taxes, *B*_*t*_ denotes the unemployment subsidy, *ρ*_*g*_ and *ρ*_*z*_ are policy smoothing parameters, *α*_*g*_ and *α*_*z*_ represent the fiscal policy response coefficients, and *v*_*t*_ represents the market value of the debt/GDP ratio.

The central bank implements a price-based monetary policy on the basis of the Taylor rule and regulates interest rates using the interest rate level, inflation, and output level of the previous period. The specific form is as follows:

15 $$\begin{gathered} \log (R_{t} ) = r_{t} = \rho_{r} r_{t - 1} + (1 - \rho_{r} )\left[ {\phi_{\pi } \log (\frac{{\pi_{t - 1} }}{\pi }) + \phi_{{\text{y}}} \log (\frac{{Y_{t - 1} }}{Y})} \right] + \tau_{r,t} \hfill \\ \tau_{r,t} = \rho_{{\varepsilon_{r} }} \tau_{r,t - 1} + \sigma_{r} \varepsilon_{r,t} \hfill \\ \end{gathered}$$

where *π* represents the inflation target, *ρ*_*r*_ represents the interest rate smoothing parameter, *φ*_*π*_ and *φ*_*Y*_ are the response coefficients of the central bank to inflation and output when the central bank implements price-based monetary policies, and *τ*_*r,t*_ represents the monetary policy shocks.

### Measurement of uncertainty risk in high-dimensional DSGE model

One of the innovations of this paper is the focus on the impact degree of the uncertainty risk of sudden shocks on macroeconomic activities. The uncertainty risk here primarily utilizes the notion defined by Basu and Bundick (2017), that is, the heteroskedastic response of the variable. Because the constructed DSGE-SV-*t* model has the Epstein–Zin preference and separability of the utility function, the higher-order moments of the endogenous state vector exhibit time-varying characteristics. Moreover, uncertainty risk information primarily exists in the third-order term of the Taylor series expansion of the DSGE model. Therefore, we can measure uncertainty risk as the difference between the third- and first-order effects of the Taylor series expansion of the DSGE model. We perform a third-order perturbation approximation to the constructed DSGE-SV-*t* model, given the continuous improvement in high-order perturbation algorithms.

16 $$\begin{gathered} x_{t + 1} = h_{x} x_{t} + \frac{1}{2}H_{xx} (x_{t} \otimes x_{t} ) + \frac{1}{6}H_{xxx} (x_{t} \otimes x_{t} \otimes x_{t} ) \hfill \\ \;\;\;\;\;\;\;\;{ + }\frac{1}{2}h_{\sigma \sigma } \sigma^{2} + \frac{3}{6}h_{\sigma \sigma x} \sigma^{2} x_{t} + \frac{1}{6}h_{\sigma \sigma \sigma } \sigma^{3} + \sigma \xi \varepsilon_{t + 1} \hfill \\ \end{gathered}$$

where *x*_*t*_ represents the third-order approximation of the macroeconomic state vector, and *ε*_*t*_ represents the structural shock vector. *h*_*x*_, *H*_*xx*_, *H*_*xxx*_, *h*_*σσ*_, *hσσx*, and *hσσσ* respectively represent the derivation of different orders of the state and shock vectors.

In the approximate system of the nonlinear DSGE model, because high-order terms tend to produce unstable steady state and explosive paths, special methods are required. The pruning method can ensure a stable path. Hence, we continue to construct the pruned state space system for the third-order approximate system. Primarily, the state variables are divided into the first-order effect *x*_*t*_^*f*^, second-order effect *x*_*t*_^*s*^, and third-order effect *x*_*t*_^*d*^. Then, the third-order effect can describe the influence degree of uncertainty risk. The state space system after pruning can be expressed as follows:

17 $$\begin{gathered} y_{t}^{d} = g_{x} (x_{t}^{f} + x_{t}^{s} + x_{t}^{d} ) + \frac{1}{2}G_{xx} ((x_{t}^{f} \otimes x_{t}^{f} ) + 2(x_{t}^{f} \otimes x_{t}^{s} )) + \hfill \\ \frac{1}{6}G_{xxx} (x_{t}^{f} \otimes x_{t}^{f} \otimes x_{t}^{f} ){ + }\frac{1}{2}g_{\sigma \sigma } \sigma^{2} + \frac{3}{6}g_{\sigma \sigma x} \sigma^{2} x_{t}^{f} + \frac{1}{6}g_{\sigma \sigma \sigma } \sigma^{3} \hfill \\ \end{gathered}$$

where *G*_*xx*_, *G*_*xxx*_, *g*_*σσ*_, *g*_*σσx*_, and *g*_*σσσ*_ represent the derivative of (*x*_*t*_, *x*_*t*_), (*x*_*t*_, *x*_*t*_, *x*_*t*_), (*σ*, *σ*), (*σ*, *σ*, *x*_*t*_), and (*σ*, *σ*, *σ*), respectively.

The impulse response function is an effective way to analyze the DSGE model. The shock effect is symmetrical for the first-order approximation, and the impulse response function has a simpler form. Given the high-order approximation, deriving the closed-form solution of the impulse response function is more complicated. The application of the pruning state space system provides convenience for deriving the impulse response function of the third-order approximated nonlinear DSGE model. The generalized impulse response function of Koop et al. (1996) is used to express the macroeconomic impulse response with uncertainty risk information in the following form:

18 $$\begin{gathered} GIRF_{{x_{t + l}^{d} }} (l,o_{i} ,(x_{t}^{f} ,x_{t}^{s} )) = \sum\limits_{j = 1}^{l - 1} {h_{x}^{l - 1 - j} } H_{xx} GIRF_{{x_{t}^{f} \otimes x_{t}^{s} }} (j,o_{i} ,(x_{t}^{f} ,x_{t}^{s} )) + \hfill \\ \sum\limits_{j = 1}^{l - 1} {h_{x}^{l - 1 - j} } \frac{1}{6}H_{xxx} GIRF_{{x_{t}^{f} \otimes x_{t}^{f} \otimes x_{t}^{f} }} (j,o_{i} ,x_{t}^{f} ) + \sum\limits_{j = 1}^{l - 1} {h_{x}^{l - 1 - j} } \frac{3}{6}h_{\sigma \sigma x} GIRF_{{x_{t}^{f} }} (j,o_{i} ) \hfill \\ \end{gathered}$$

where *l* represents the number of periods of the impulse response, and *o*_*i*_ is the magnitude of shock *i*.

### Estimation method of high-dimensional DSGE model

The DSGE-SV-*t* model assumes that the exogenous shocks in our model are non-Gaussian. The model uses the Student’s *t*-thick tail distribution to describe the extreme risk caused by the suddenness of exogenous shocks and considers the stochastic volatility of the shock process. Regarding the model’s estimation method, although the random block Metropolis–Hastings algorithm is relatively time-consuming, it can improve the efficiency of posterior sampling. Given the sample data *y*_1:*T*_ during *t* = 1,…, *T*, the model parameters Θ = (Θ^*S*^, Θ^*V*^), the latent variable *λ*_1:*T*_ used to construct the Student-*t* shock, and the nonlinear latent variable *h*_1:*T*_ representing time-varying volatility process, the Markov chain Monte Carlo method can be used to sample the posterior distribution of the parameters of the high-dimensional DSGE model.

19 $$\pi (\Theta ,\lambda_{1:T} ,h_{1:T} \left| {y_{1:T} } \right.) \propto f(y_{1:T} ,\lambda_{1:T} ,h_{1:T} ) \cdot \pi (\Theta ) \cdot 1\left\{ {\Theta \in D} \right\}$$

where *π*(Θ) represents the prior distribution, *f*(*y*_1:*T*_, *λ*_1:*T*_, *h*_1:*T*_) represents the likelihood function, and 1{Θ ∈ *D*} is the indicator function, the value of which is 1 when the parameter Θ is in the certain domain *D* and 0 otherwise.

### Parameter estimation

Before performing posterior sampling on the DSGE-SV-*t* model, the prior distribution of some parameters needs to be specified in advance. For the high-dimensional DSGE model, the construction of a suitable prior distribution involves mapping the structural parameters into the state space form, which increases the complexity. We use a two-step sampling method to set the prior distribution in this paper and minimize the influence of prior specification errors on the posterior distribution. Primarily, a standard prior distribution is set for the structural parameters, and we sample 10,000 times for the joint prior and match with the implicit distribution of each moment of the data. Then, the RB-MH algorithm is used to estimate the first 20 observations of the DSGE-SV-*t* model to obtain a prior distribution close to the actual data.

Then, we follow Eq. (19) to sample the posterior distribution. First, the RB-MH algorithm is employed to extract the structural parameter Θ^*S*^ from the conditional posterior *π*(Θ^*S*^ | *y*_1:*T*_, Θ^*V*^, *λ*_1:*T*_, *h*_1:*T*_). Second, Gibbs sampling is performed on the non-Gaussian latent variables *h*_1:*T*_ and *λ*_1:*T*_ and the volatility parameter Θ^*V*^ from the conditional posterior *π*(Θ^*V*^, *λ*_1:*T*_, *h*_1:*T*_ |*y*_1:*T*_, Θ^*S*^). In particular, the sampling of the conditional density *π*(*λ*_1:*T*_| *y*_1:*T*_, Θ, *h*_1:*T*_) adopts a mixed normal expression based on the Student’s *t*-distribution. We iterate this process to convergence to obtain the joint posterior distribution. This method is applied to the remaining quarterly data to estimate the high-dimensional DSGE model. The RB-MH algorithm has high efficiency and does not need to set too large sampling times. Here we set 11,000 simulation times and a burn-in period of 1000 times.

Because China has achieved world-renowned victories in the fight against the COVID-19, and the sample period selected in this study includes this pandemic, using Chinese data for the analysis is typical. We select output, consumer price index, investment, interest rates, employment, and government spending from the first quarter of 2005 to the second quarter of 2020. The data are from the website of the National Bureau of Statistics in China, the website of the People’s Bank of China, the Wind database, the CEIC China Economic Database, and the macroeconomic data set of Chang et al. (2016). Trend and seasonal factors are in the observed values of the variables. The logarithm of the observed values of all of the variables is first taken using the X11 method used for seasonal adjustments, except for the interest rate variables. Then, the HP filter is utilized to eliminate the long-term trend. We follow An and Schorfheide (2007) and set the prior distribution of stochastic shock-related parameters. Posterior sampling is performed using Eq. (19) and the Metropolis–Hastings algorithm.

The household sector’s discount factor is 0.99 to ensure that the steady-state value of the model is as close to the actual result as possible, in reference to the specification of most of the literature. The quarterly value of the capital depreciation rate is 0.024, which is equivalent to an annual depreciation rate of 10%. In particular, measuring the number of employed population refers to Besma (2015). In addition, the share of capital in output is 0.36, and the proportion of capital to output is calculated using China’s real fixed capital and real GDP during the sample period. The share of government expenditures in the national economy is 20.56%. Table 1 displays the marginal prior distribution confidence interval and the mean value of the posterior distribution of the structural parameters. Table 2 shows the marginal prior distribution confidence interval and the mean value of the posterior distribution of the volatility parameter. The volatility parameters primarily include technology, risk preference, and government expenditure shocks, which are represented by subscripts *a*, *b*, and *g*, respectively. The parameter estimation results of risk preference show that agents have a risk-averse attitude toward future uncertainty. Moreover, the estimated coefficient value of the degree of freedom is also low, indicating that the tail risk is caused by the impact of a sudden event.

**Table 1:** Bayesian estimation results of structural parameters

Parameter	Meaning	Prior distribution	Prior confidence interval	Posterior mean
*γ*	Investor's attitude towards uncertainty	Normal	(0.82, 0.99)	0.84
*σ*_*L*_	Inverse of Frisch's elasticity	Gamma	(1.55, 2.55)	1.97
*b*	Habitual smoothing factor	Uniform	(0.98, 0.99)	0.99
*σ*_*C*_	Reciprocal of the elasticity of intertemporal substitution	Uniform	(1.21, 1.86)	1.54
*κ*	Capital utilization	Beta	(0.11, 0.31)	0.22
*s*	Investment adjustment cost	Normal	(5.11, 9.12)	6.97
*m*_*p*_	Price stickiness	Beta	(0.72, 0.95)	0.75
*m*_*w*_	Wage stickiness	Beta	(0.71, 0.89)	0.71
*ι*	Degree of price indexation	Beta	(0.31, 0.43)	0.39
*χ*_*w*_	Degree of wage indexation	Beta	(0.02, 0.11)	0.07
*φ*_*π*_	Response coefficient of interest rate to inflation	Normal	(1.51, 2.53)	2.26
*φ*_*y*_	Response coefficient of interest rate to output volatility	Normal	(0.11, 0.22)	0.13
*ρ*_*r*_	Continuity of monetary policy	Beta	(0.57, 0.61)	0.59
*ρ*_*g*_	Continuity of fiscal expenditure	Beta	(0.45, 0.47)	0.46
*ρ*_*z*_	Continuity of government transfer	Beta	(0.43, 0.49)	0.48
*α*_*g*_	Fiscal expenditure response coefficient	Normal	(0.18, 0.28)	0.24
*α*_*z*_	Government transfer response coefficient	Normal	(0.14, 0.24)	0.21

**Table 2:** Bayesian estimation results of volatility parameters

Parameter	Prior confidence interval	Posterior mean	Parameter	Prior confidence interval	Posterior mean
*μ*_*a*_	(-0.52, 0.12)	− 0.23	*φ*_*g*_	(0.71, 0.74)	0.72
*μ*_*b*_	(3.82, 5.36)	4.74	*φ*_*z*_	(0.72, 0.75)	0.73
*μ*_*g*_	(0.62, 1.01)	0.88	*σ*_*a*_^2^	(0.01, 0.04)	0.02
*μ*_*z*_	(1.01, 2.18)	1.33	*σ*_*b*_^2^	(0.01, 0.03)	0.01
*φ*_*a*_	(0.71, 0.79)	0.74	*σ*_*g*_^2^	(0.01, 0.04)	0.02
*φ*_*b*_	(0.71, 0,78)	0.72	*σ*_*z*_^2^	(0.01, 0.04)	0.02

## Empirical research

### Analysis of the dynamic effects of macroeconomics

We extended the New Keynesian model and constructed a DSGE-SV-*t* model that includes heavy-tail structural shocks and time-varying volatility. We adopt the third-order perturbation method to solve the model, and the impulse response of macroscopic variables is calculated. This section adopts this approach and focuses on analyzing the dynamic effects of consumption, investment, employment, inflation, interest rates, and fiscal expenditures, given the influence of uncertainty risk. Subsequently, we compute the dynamic response of macro variables given technological, fiscal policy, and investor risk preference shocks, respectively. Our paper is distinguished from other studies because it measures the impact of uncertainty risk on the macroeconomic transmission mechanism. Specifically, the impact of uncertainty risk on macroeconomic transmission channels can be measured using the difference between the impulse response obtained by the third- and first-order approximations.

Because investors pay more attention to uncertainty risk in the economic recession period than in the economic boom period, we further analyze the impact of uncertainty risk on the transmission mechanism of macro variables given different inflation states and different economic conditions. We respectively analyze the impact of uncertainty risk on the economic transmission mechanism caused by fiscal policy and risk preference shocks during periods of high and low inflation. We also analyze the impact of uncertainty risk on the economic transmission mechanism caused by fiscal policy and risk preference shocks during periods of economic prosperity and recessions.

### Macroeconomic dynamic effects of technological shocks under uncertainty risk

**Fig. 1:**
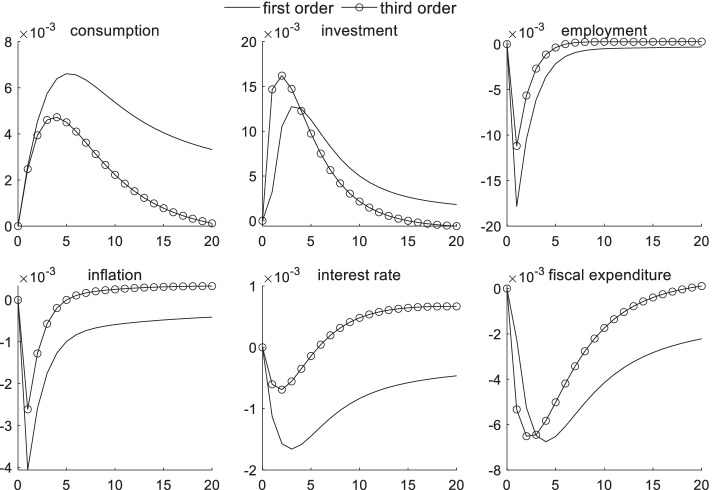
Macroeconomic effects of technological shocks under uncertainty risk

Figure 1 shows the dynamic effects of various macroeconomic variables after one unit of a positive technological shock. Judging from the overall dynamic trend of the first- and third-order effects, positive technological shocks have brought economic vitality into the entire market, resulting in an increase in consumption and investments. Total factor productivity increases given a positive technological shock. Moreover, manufacturers have chosen to increase investments and expand their output scale to optimize their profits, which are accompanied by an increase in investment levels. Affected by traditional labor being replaced with emerging technologies, such as artificial intelligence and big data, the employment rate has declined sharply in the short term. However, it gradually returned to normal over time. Given the strong development of financial technology, emerging financial forms, such as Internet finance, have eased the financing constraints of small and medium-sized enterprises to a certain extent, causing interest rates to decline in the short term. As interest rates decline, households choose to increase their consumption. Positive technological shocks have played a role in curbing inflation and fiscal expenditures in the short term because technological advancements have reduced manufacturers’ marginal costs. Positive technological shocks have improved the overall social welfare situation and have reduced fiscal expenditures. The suddenness of uncertainty risk causes fiscal expenditures to fail to be fully included in the government’s rescue budget, and price information is lacking, which creates negative fluctuations in fiscal expenditures.

A comparison of linear and third-order effects shows that uncertainty risk has a significant impact on the transmission channels of various macroeconomic sectors. A description of the heterogeneity of uncertainty risk in different economic states is detailed in the following sections. In Fig. 1, uncertainty risk has the strongest impact on employment and consumption channels. The impact of uncertainty risk on consumption channels continues to increase after the second period and reaches a maximum during the 20th period. Given investors’ risk aversion, they are more cautious about consumption during technology shocks related to sudden uncertain risks. In addition, the negative impact on employment caused by technological shocks is deeper in the short term, with the difference reaching 0.7% in the first period. The impact gradually weakens. The impact on the inflation transmission mechanism is also most obvious in the first period and then gradually weakens. As investors quickly capture the central bank’s expected attitude toward sudden uncertain risks, the investment scale expands more rapidly in the short term. The minimal impact of uncertainty risk on interest rates indicates that the central bank includes part of the risk information when implementing monetary policy, and its expected management effect is better. When uncertain risks are anticipated, the negative impulse response of fiscal expenditures becomes smaller, reflecting the effective response measures taken by government departments during public emergencies.

### Macroeconomic effects of fiscal policy shocks under uncertainty risk

**Fig. 2:**
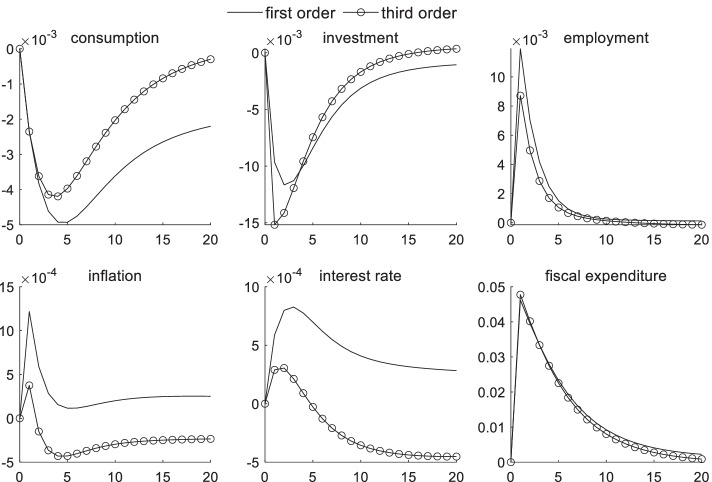
Macroeconomic effects of fiscal policy shocks under uncertainty risk

Figure 2 illustrates the dynamic effects of various macroeconomic variables after one unit of a positive fiscal expenditure shock. As observed from the general trend of the first- and third-order effects, the positive fiscal policy shock produced a crowding-out effect on private consumption and investment because the issuance of local government bonds by government agencies through local financing platforms reduces the liquidity in the market, which increases interest rates and inhibits consumption and investments. In theory, investments represent a decreasing function of interest rates; when interest rates increase, investments decrease. Monopolistic competition manufacturers reduce production and increase prices to maximize profits and avoid sudden risks. The government raises taxes to finance its expenditures, which reduces private income and private consumption and investments. Moreover, a household sector with rational expectations chooses to reduce consumer spending. The government’s transfer payments to promote employment have had the effect of improving short-term employment. An increase in government spending causes price levels to increase, which increases the level of inflation in the short term. Moreover, according to the Phillips curve theory, the unemployment rate decreases when inflation increases.

A comparison of the first- and third-order effects shows that, in the presence of uncertainty risk, manufacturers further reduce their investments and output scale in the first four periods to avoid sudden risks. In this case, the crowding-out effect of fiscal expenditures on investments is stronger. However, the crowding-out effect of fiscal expenditures on consumption and investment is weaker after four periods. Investments can quickly return to normal if the economic situation improves and expected management strengthens. The impact of uncertainty risk on household consumption begins to appear in the third period and reaches a maximum in the 20th period, with a difference of 0.2%. After considering the uncertainty risk factors, the crowding-out effect of fiscal policy stimulus on consumption gradually weakens from the third period, and the crowding-out effect on private investment gradually weakens from the fifth period, causing relatively small short-term inflation effects. The impulse response fluctuation of the first-order approximation is always greater than that of the third-order approximation. The third-order approximation method is observed to better avoid the explosion path generated in the solution.

### Macroeconomic effects of risk preference shocks under sudden uncertainty

**Fig. 3:**
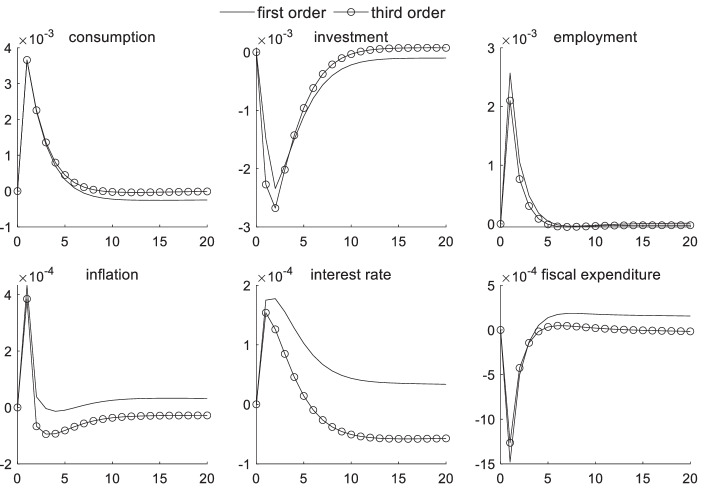
Macroeconomic effects of risk preference shock under sudden uncertainty

Agents’ risk-averse attitudes indicate that the public expects to resolve future uncertainties as soon as possible to reduce the risk of sudden uncertainties. Figure 3 illustrates the impact of risk preference shocks on the transmission mechanism of macroeconomic variables under the influence of sudden uncertainty. Judging from the overall trend in the first- and third-order effects as a result of risk-averse attitudes, agents reduce the investment scale considering sudden uncertain risks. After the occurrence of sudden uncertain events, investors hold more cash as a preventive motive, causing interest rates to increase in the short term. The increase in interest rates also further reduce investments in the corporate sector, causing negative investment volatility. The risk-averse household sector consumes more in the current period when considering the uncertainty of future consumption costs. Additionally, they give up leisure opportunities to increase employment. A decrease in investments and an increase in consumption causes a shortage in the supply of final products in the market, and an increase in product prices triggers an increase in inflation. The government often substantially adjusts fiscal expenditures in a short period to reduce unnecessary fiscal expenditures and address the risk of future uncertainty.

The difference between linear and third-order effects reflects the impact of uncertainty risk on macroeconomic transmission influenced by a risk preference shock. By comparison, uncertainty risk can explain 22% of the decline in investments, 13% of the increase in interest rates, and 16% of the increase in employment rates, given a risk preference shock. Because investors have Epstein-Zin preferences, this parameter reflects the degree of agents’ aversion to the increase in future uncertainty. Given the uncertainty risk factor, the degree of the impulse response to inflation and interest rates during risk preference shocks has been reduced, whereas the impact on the transmission mechanism of consumption and employment is relatively weak.

After a comprehensive comparison of the macroeconomic dynamic effects during technological, fiscal policy, and risk preference shocks, macroeconomic impulse responses under linear and third-order effects are found to be correlated in the overall variation trend. This correlation also verifies the robustness of the method constructed in our model. Further, uncertainty risk generates a stronger impact on the transmission channels of investment and employment. The uncertainty risk shown by the third-order effect can change the transmission mechanism of macroeconomic variables (Andreasen 2013), but this change gradually returns to the steady state in the long term. We respectively change the volatility parameters for technology, risk preference, and government spending shocks from 0.7 to 0.8 to examine differences in the empirical results. The empirical results only change slightly within the sensitivity analysis interval of the parameters, which has a minimal impact on the conclusions.

### Macroeconomic effects of fiscal policy and risk preference in different economic environments

Given different circumstances, such as different inflation levels and economic conditions, macroeconomic variables display heterogeneity. Influenced by the sudden uncertain risk represented by the COVID-19 pandemic, real economic activities experienced a period of depression. During an economic downturn, consumption and investment levels are usually low, and the marginal effect of additional consumption and the marginal benefit of additional investment are usually higher than those during a period of economic prosperity. When inflation is high, nominal rigidity is usually detrimental to enterprises because they typically cannot reach the optimal price. Therefore, we further analyze the macroeconomic dynamic effects of uncertainty risk under different circumstances that are influenced by agents’ fiscal policy and risk preference shocks, respectively.

### Macroeconomic effects of fiscal policy shocks under uncertainty risk

**Fig. 4:**
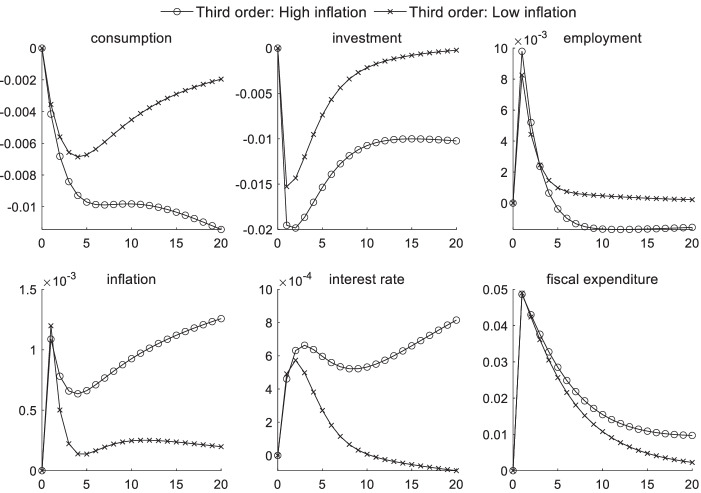
Impact of uncertainty risk on fiscal policy shocks under different inflation scenarios

**Fig. 5:**
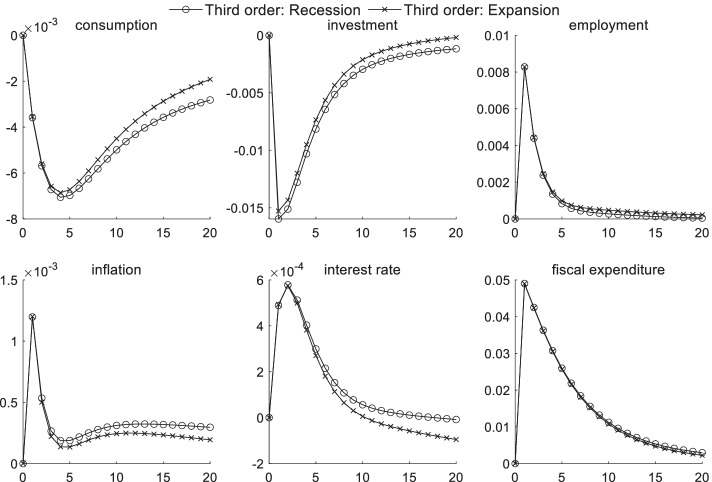
Impact of uncertainty risk on fiscal policy shocks in different economic conditions

Figures 4 and 5 respectively display the impact of uncertainty risk on fiscal policy shocks under different inflation states and different economic conditions. When the inflation level is high, the impulse response intensity of consumption, investments, and interest rates are all higher than that at a low inflation level. Regardless of the type of inflation periods or economic environments, a positive fiscal policy shock generates a certain crowding-out effect on consumption and investments. This effect is more pronounced during periods of high inflation, whereas the numerical difference is not obvious in different economic conditions. The results of the parameter estimation also show that investors’ risk preference is risk-averse. Moreover, when faced with the uncertainty of future expectations, investors hold pessimistic attitudes toward future expected returns, further reducing the willingness to invest.

When uncertainty risk is considered, the increase in market interest rates caused by government agencies’ debt issuances through local financing platforms becomes more pronounced during periods of high inflation—causing a series of chain reactions. The chain reaction in high inflation periods is larger than that in low inflation periods. As interest rates continue to increase, the investment scale declines even more, and the product price continues to increase. This effect further aggravates inflation, and consumers’ desire to purchase also becomes weaker, significantly reducing the effect of government transfer payments to improve employment during high inflation periods. Curbing inflation is viewed as becoming an important goal of regulatory authorities. In an economic downturn, reducing the unemployment rate is often the main policy target for monetary authorities, in addition to stimulating the economy, thus posing a significant challenge to the coordination of the central bank’s monetary and fiscal policies given macroprudential supervision. From the perspective of the influence of uncertainty risk on fiscal policy shocks in different economic conditions, uncertainty risk has a minimal short-term impact on macroeconomic transmission mechanisms given fiscal policy shocks.

### Macroeconomic effects of risk preference shocks under sudden uncertainty

**Fig. 6:**
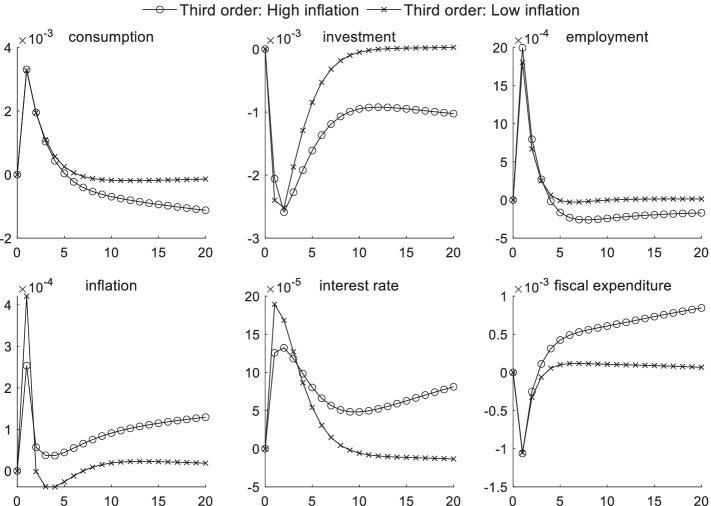
Impact of uncertainty risk on risk preference shocks under different inflation scenarios

**Fig. 7:**
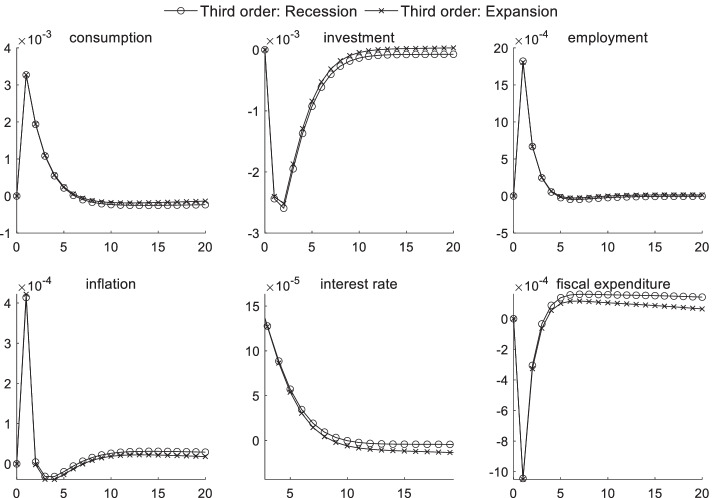
Impact of uncertainty risk on risk preference shocks under different economic conditions

Figures 6 and 7 show the volatility state of the macroeconomic variables that are affected by a positive risk preference shock given different inflation and economy states and sudden uncertainty. The inflation factor generates a stronger impact on macroeconomic transmission than does the external economic condition factor, as observed from the overall impact of uncertainty risk on macroeconomic transmission. In different inflation periods, the influence of a risk preference shock on consumption, investments, and employment begins to appear in the third period. During the first three periods, the preventive consumption increase caused by one unit of a positive risk preference shock reaches up to 0.34%, and the short-term increase in the employment rate reaches 0.2% in the second period. However, different economic environments generate minimal differences in the influence of macroeconomic transmission channels during a risk preference shock considering the sudden uncertain risk factor.

During high inflation periods, the authorities readjust expenditures from the second period after the occurrence of sudden uncertain events, and the control willingness of high inflation is greater than that in a low inflation period. Because agents have Epstein–Zin preferences, indicating that they hate the risk of future uncertainties and are pessimistic about expected marginal future returns, the investment scale declines. Moreover, this influence channel is more pronounced during high inflation periods.

High inflation reduces the consumption capacity of the household sector from the third period, relative to low inflation, because of the effect of uncertainty risk. In the first three periods, consumption is not affected by inflation because of a preventive motive. According to the Phillips curve, the employment rate is lower in a high inflation environment given the same conditions. Additionally, a high inflation environment inhibits the transmission of interest rates in the macroeconomy during the first three periods. On the whole, the volatility of fiscal department expenditures is most affected by the external economic environment, and fiscal policy often requires three periods of strategic adjustments after unexpected events occur. Hence, higher requirements for the financial supervision sector are required to more promptly address crisis events under macroprudential supervision.

## Conclusion

This paper innovatively constructs a high-order approximation DSGE model that includes the characteristics of a thick-tailed distribution and stochastic volatility shocks to investigate the impact of uncertainty risk on the transmission channel among macroeconomic sectors and to better analyze the dynamic effect of uncertainty risk on macroeconomics. The RB-MH algorithm is employed to estimate the high-dimensional DSGE model. Then, a series of empirical analyses is carried out on 2005 to 2020 data from China. Although the random block method can improve posterior estimation efficiency, further improving this efficiency in high-dimensional DSGE models is still an important issue for future research.

The impulse response analysis using the DSGE-SV-*t* model shows that investors are more cautious about consumption during technology shocks with uncertainty risk because of their risk aversion. In addition, the negative impact on employment caused by technology shocks is stronger in the short term. This paper found that when uncertainty risk factors are taken into account, the crowding-out effect of a fiscal policy stimulus on consumption and private investment is weakened—unlike the findings in previous studies—and the short-term inflation effect is relatively small. However, this crowding-out effect is even more obvious during high inflation periods. Moreover, the effect of transfer payments adopted by the government to improve employment during high inflation periods is not obvious. This paper also found that the degree of the impulse response to inflation and interest rates given risk preference shocks affected by sudden uncertainty is reduced, and the impact on the transmission of consumption and employment is relatively weak. These findings indicate that curbing inflation should be an important goal of regulatory authorities.

After the occurrence of sudden uncertainty events, fluctuations in government expenditures are most affected by the external economic environment. The research in this paper provides a reference for regulatory authorities to enhance their ability to resist sudden risks and emergencies. Public finance departments should fully consider the impact of various external environments when coordinating and collaborating with monetary policy to carry out macrocontrols. Fully clarifying the expected adjustment objectives of the macroeconomy and fully integrating macro and micro measures are necessary. At the same time, the central bank and public finance departments should actively explore new control strategies to ensure that coordinated prudential policies can be comprehensively used in the event of emergency economic shocks.

## Data Availability

The datasets used are available from the corresponding author upon reasonable request.

## Abbreviations

DSGE: Dynamic stochastic general equilibrium
DSGE-SV: Dynamic stochastic general equilibrium model with stochastic volatility
DSGE-SV-*t*: Dynamic stochastic general equilibrium model with stochastic volatility and heavy tailed distribution

## Footnotes

1 Chib S, Shin M, Tan F (2021) DSGE-SVt: An econometric toolkit for high-dimensional DSGE models with SV and t Errors. Comput Econ doi: org/10.1007/s10614-021–10 200-y.
